# Supplementary material for: Acoustofluidic Interfaces for the Mechanobiological Secretome of MSCs
Source: Nat Commun. 2023 Nov 22;14:7639. doi: 10.1038/s41467-023-43239-6 (PMC10665559; doi:10.1038/s41467-023-43239-6)
Supplement: Supplementary file 1 — Supplementary information [file 41467_2023_43239_MOESM1_ESM.pdf]

Supplementary Information

AIMS: Acoustofluidic Interfaces for the Mechanobiological Secretome of MSCs

Ye He[1], Shujie Yang[1], Pengzhan Liu[1], Ke Li[1], Ke Jin[1], Ryan Becker[2], Jinxin Zhang[1], Chuanchuan Lin[3], Jianping Xia[1], Zhehan Ma[2], Zhiteng Ma[1], Ruoyu Zhong[1], Luke P. Lee[4,5,6*], and Tony Jun Huang[1*]

[1]Thomas Lord Department of Mechanical Engineering and Materials Science, Duke University, Durham, NC 27708, USA

[2]Department of Biomedical Engineering, Duke University, Durham, NC 27708, USA

[3]Department of Blood Transfusion, Irradiation Biology Laboratory, Xinqiao Hospital, Chongqing, 400037, China

[4]Harvard Medical School, Harvard University; Renal Division and Division of Engineering in Medicine, Department of Medicine, Brigham and Women's Hospital, Boston, MA 02115, USA

[5]Department of Bioengineering, Department of Electrical Engineering and Computer Science, University of California, Berkeley, Berkeley, CA 94720, USA

[6]Department of Biophysics, Institute of Quantum Biophysics, Sungkyunkwan University, Suwon, Korea

* Corresponding authors: L.P.L and T.J.H. (email: lplee@bwh.harvard.edu; tony.huang@duke.edu)

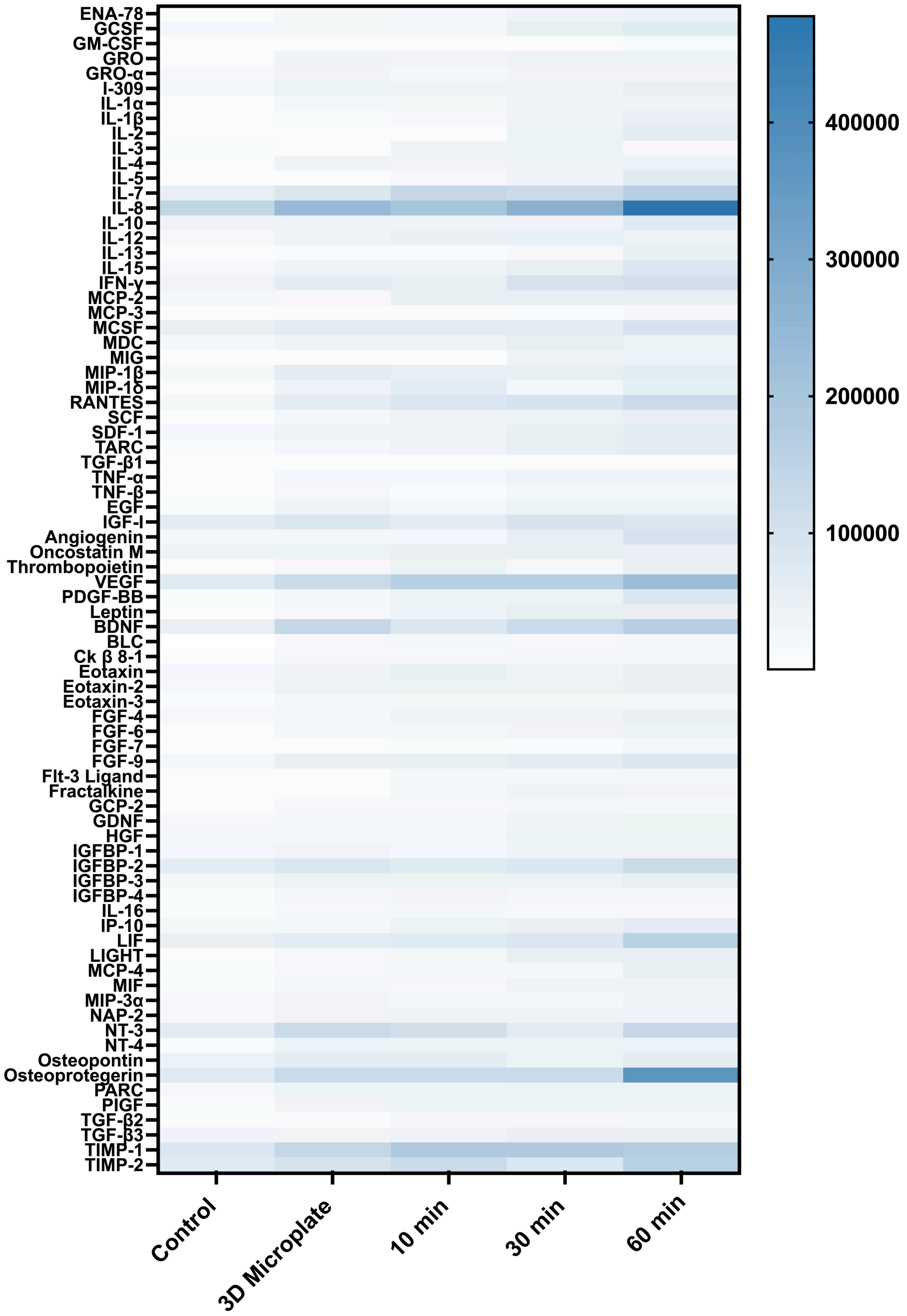

Supplementary Figure 1 | Heatmap presenting the integrated densities of different groups after incubating with the cytokine array for 4 days (without Z-score normalization and clustering process). Source data are provided as a Source Data file.

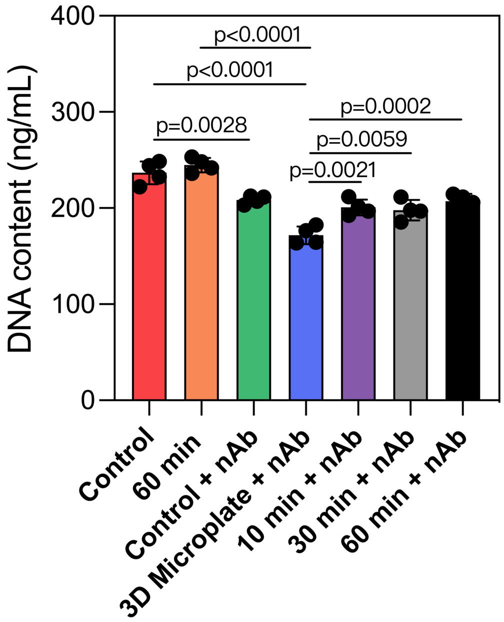

Supplementary Figure 2 | CyQUANT™ Cell Proliferation Assay measuring the DNA contents after functional blocking of N-cadherin of MSCs and incubating for 4 days. Data are graphed as the mean ± SD (n=4, biological repeats). The statistical analysis was performed using one-way ANOVA with Tukey's post-hoc test by GraphPad Prism 9.0. Source data are provided as a Source Data file.

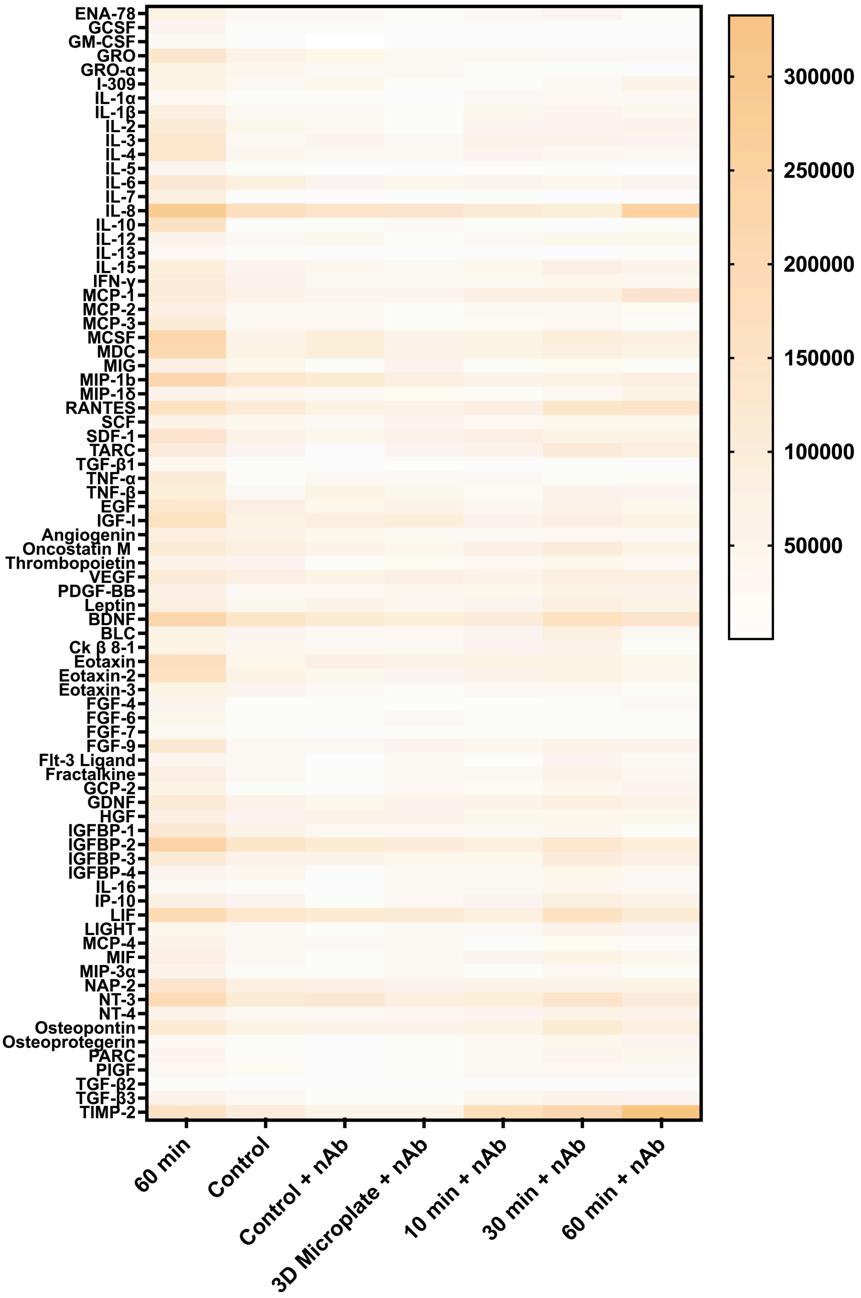

Supplementary Figure 3 | Heatmap presenting the integrated densities of different groups after functional blocking N-cadherin of MSCs and incubating with the cytokine array for 4 days (without Z-score normalization and clustering process). Source data are provided as a Source Data file.

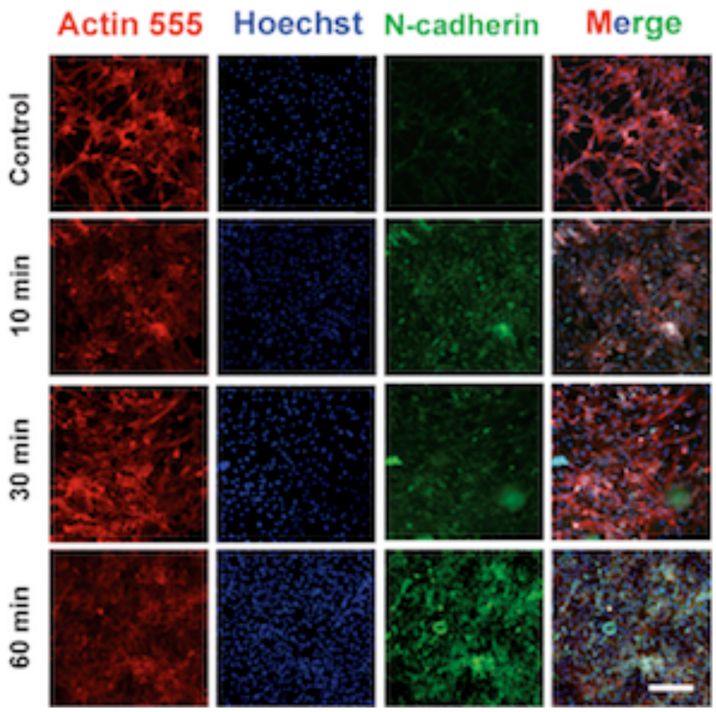

Supplementary Figure 4 | Representative immunofluorescence images of different groups after incubating for 3 days. The expression of N-cadherin is shown in green. The cytoskeleton is shown in red. The cell nucleus is shown in blue. n=3 tests. Scale bar: 50 μm.

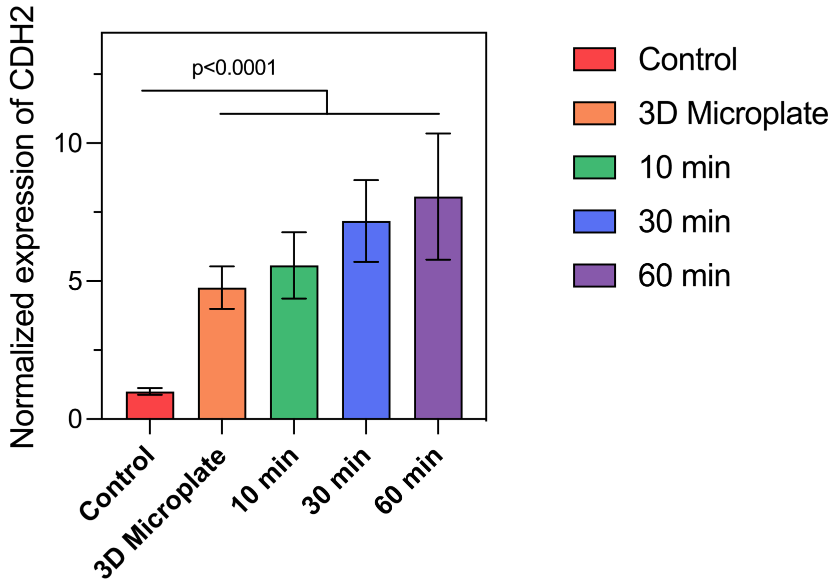

Supplementary Figure 5 | QPCR measuring the relative expression of CDH2 gene (N-cadherin) of different groups after incubating for 3 days. Data are graphed as the mean ± SD (n=3, biological repeats). The statistical analysis was performed using one-way ANOVA with Tukey's post-hoc test by GraphPad Prism 9.0. Source data are provided as a Source Data file.

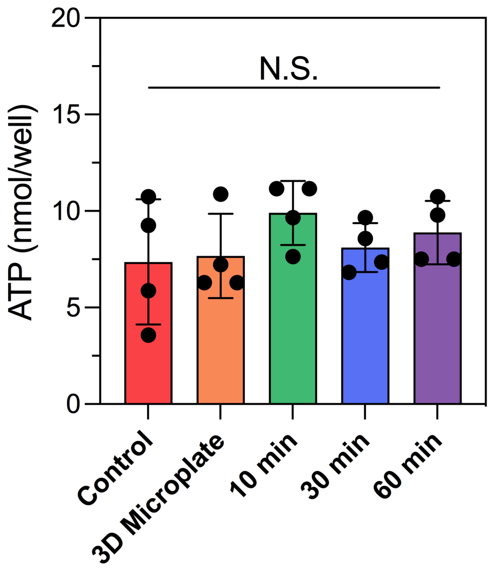

Supplementary Figure 6 | The analysis of ATP concentrations of different groups after incubating for 4 days. Data are graphed as the mean ± SD (n=4, biological repeats). The statistical analysis was performed using one-way ANOVA with Tukey's post-hoc test by GraphPad Prism 9.0. Source data are provided as a Source Data file.

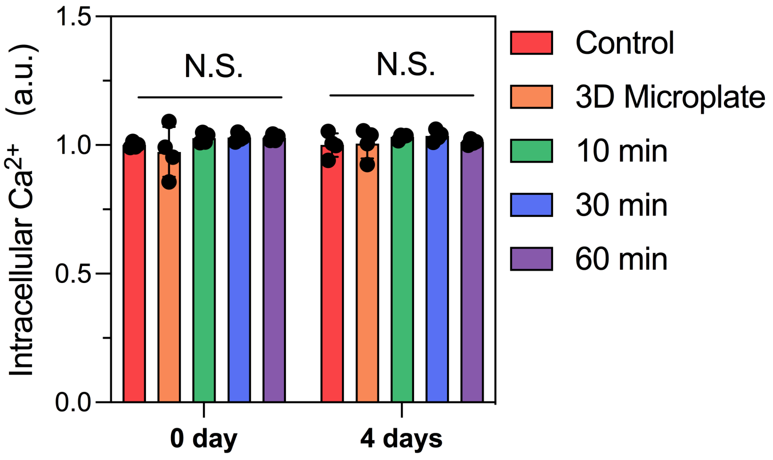

Supplementary Figure 7 | The analysis of intracellular Ca^{2+} concentrations of different groups after incubating for 4 days. Data are graphed as the mean ± SD (n=4, biological repeats). The statistical analysis was performed using one-way ANOVA with Tukey's post-hoc test by GraphPad Prism 9.0. N.S.: not significant. Source data are provided as a Source Data file.

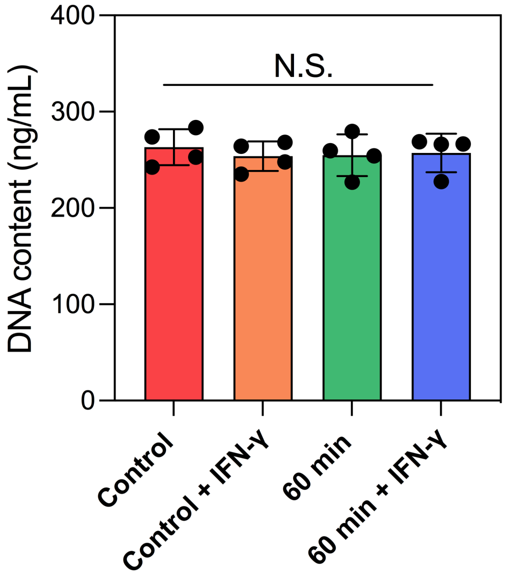

Supplementary Figure 8 | CyQUANT™ Cell Proliferation Assay measuring the DNA contents of IFN-γ primed MSCs from different groups and incubating for 4 days. Data are graphed as the mean ± SD (n=4, biological repeats). The statistical analysis was performed using one-way ANOVA with Tukey's post-hoc test by GraphPad Prism 9.0. N.S.: not significant. Source data are provided as a Source Data file.

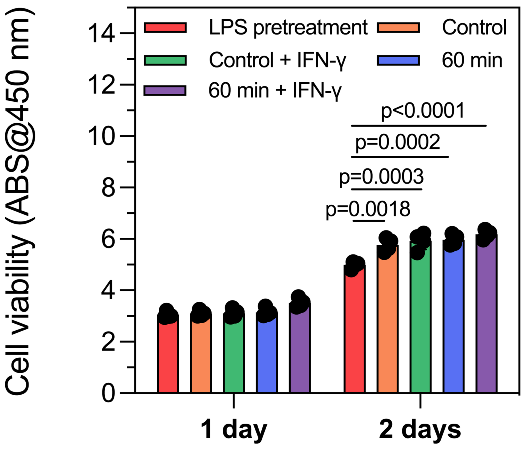

Supplementary Figure 9 | CCK-8 assay measuring the viability of Human THP-1 cells from different groups after incubating for 1 and 2 days. Data are graphed as the mean ± SD (n=4, biological repeats). The statistical analysis was performed using one-way ANOVA with Tukey's post-hoc test by GraphPad Prism 9.0. Source data are provided as a Source Data file.

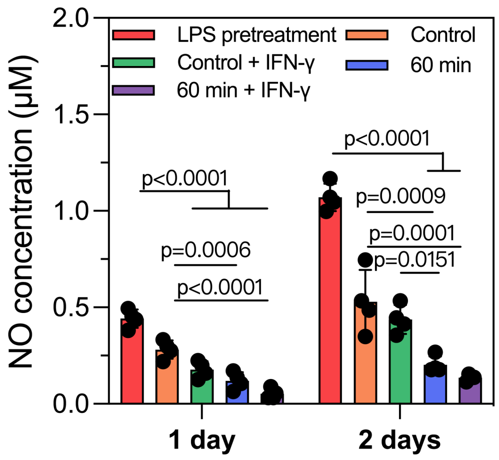

Supplementary Figure 10 | The analysis of nitric oxide (NO) production of different groups after incubating for 1 and 2 days. Data are graphed as the mean ± SD (n=4, biological repeats). The statistical analysis was performed using one-way ANOVA with Tukey's post-hoc test by GraphPad Prism 9.0. Source data are provided as a Source Data file.

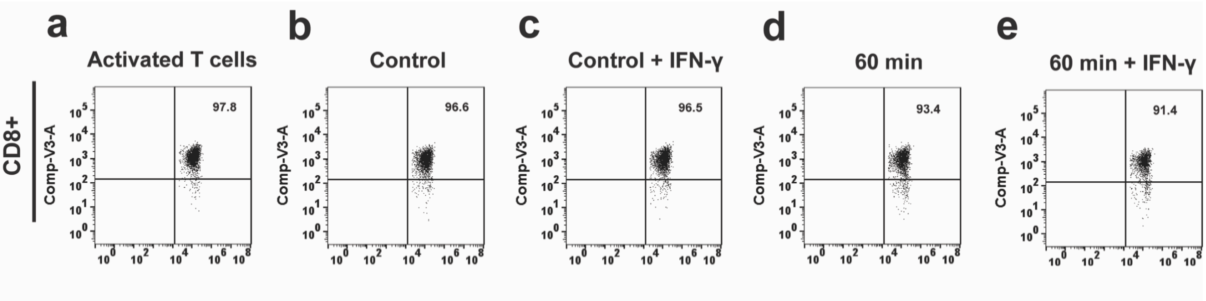

Supplementary Figure 11 | Flow cytometry analysis measuring the percentage of CD8 positive T cells of each group after incubating with the conditional medium from MSCs for 3 days. a: Activated T cells, b: Control, c: Control + IFN-γ, d: 60 min, e: 60 min + IFN-γ. Source data are provided as a Source Data file.

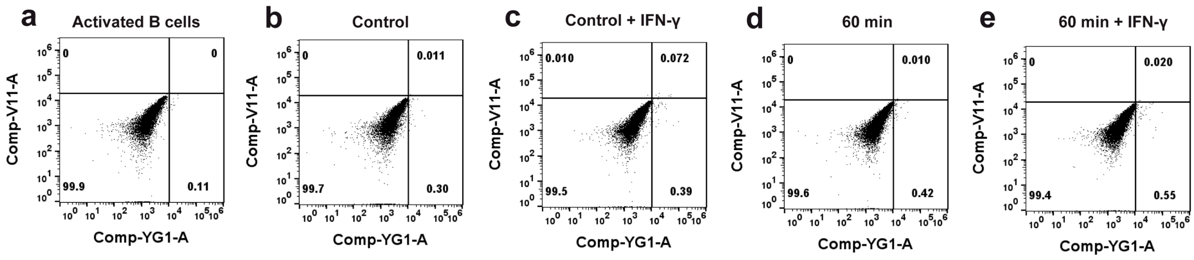

Supplementary Figure 12 | Flow cytometry analysis measuring the B cell apoptosis of each group after incubating with the conditional medium from MSCs for 7 days. a: Activated T cells, b: Control, c: Control + IFN-γ, d: 60 min, e: 60 min + IFN-γ. Source data are provided as a Source Data file.

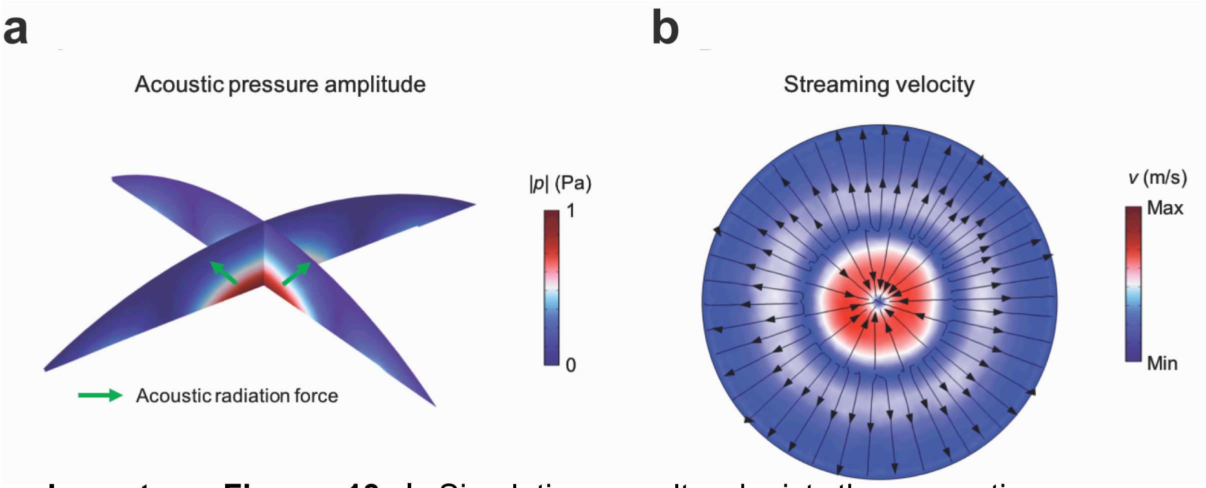

Supplementary Figure 13 | Simulation results depict the acoustic pressure amplitude **(a)** and acoustic streaming velocity **(b)** on a cross-sectional plane near the bottom of the device.

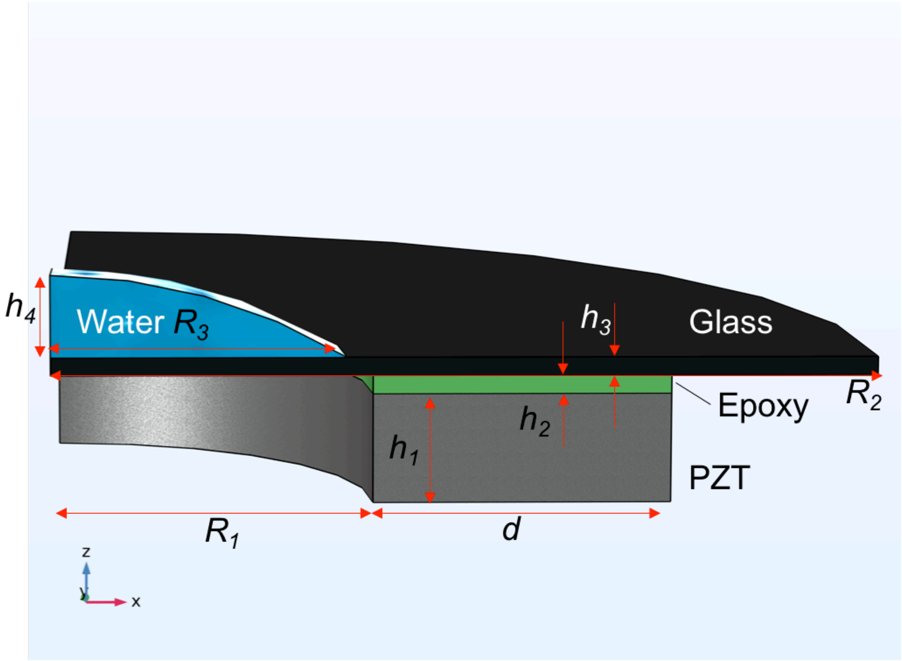

Supplementary Figure 14 | Illustration of the geometry and dimensions of the simulation model. A fully coupled three-dimensional model was developed by coupling the acoustic pressure module and solid mechanics module to calculate the acoustic intensity distribution. The model setup consisted of a ring piezoelectric transducer (PZT) located at the base of a glass substrate, affixed with a thin layer of Epoxy. Positioned atop this glass substrate was a small droplet. The geometric parameters of the model can be find in Table 1 (main text).
