# Supplementary figures and images for: Acoustofluidic Interfaces for the Mechanobiological Secretome of MSCs
Source: Nat Commun. 2023 Nov 22;14:7639. doi: 10.1038/s41467-023-43239-6 (PMC10665559; doi:10.1038/s41467-023-43239-6)

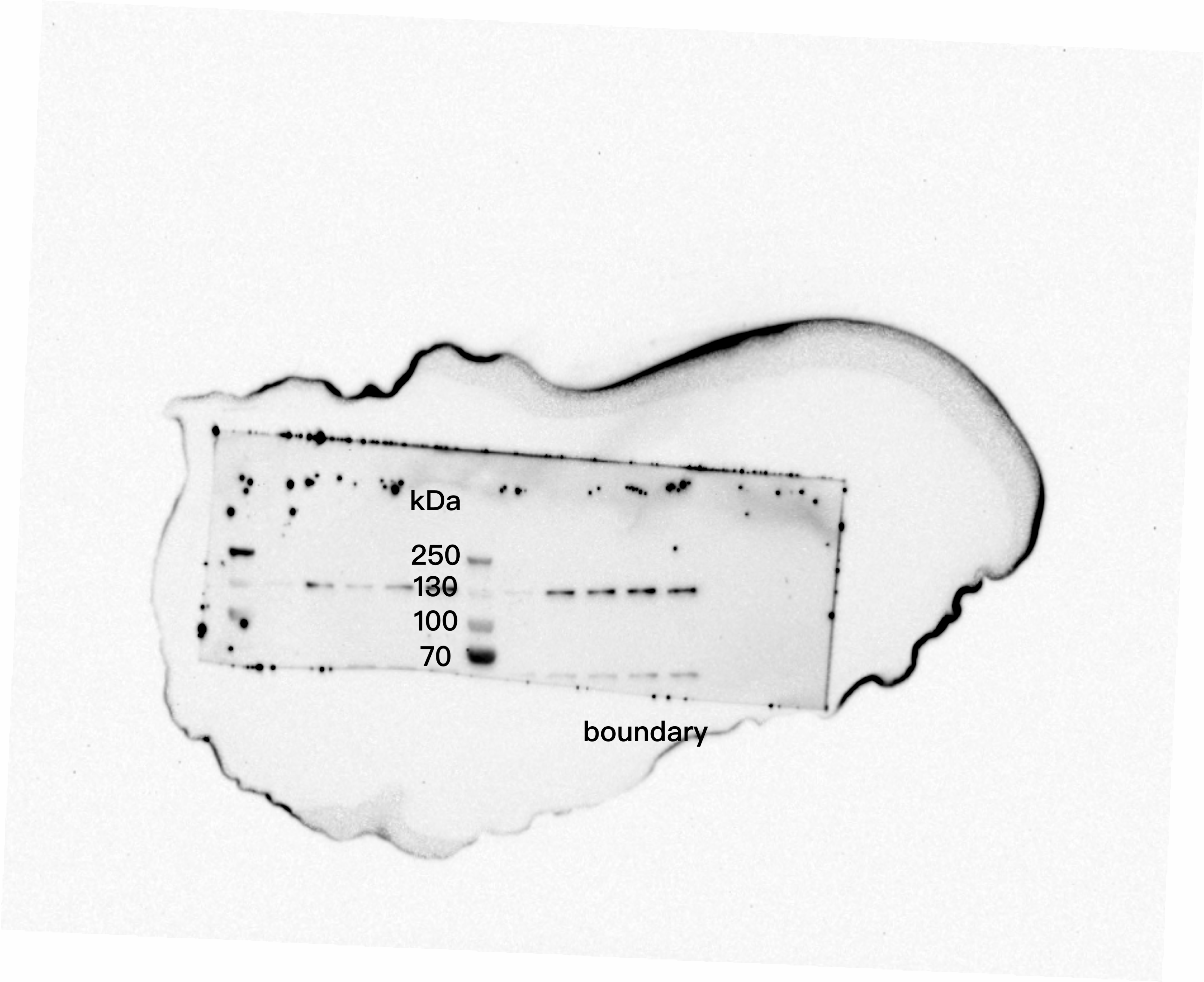

Supplement: Supplementary file 4 — Source data [file 41467_2023_43239_MOESM4_ESM.zip › Source data/Western blot original image /N-cadherin (right half).tif]

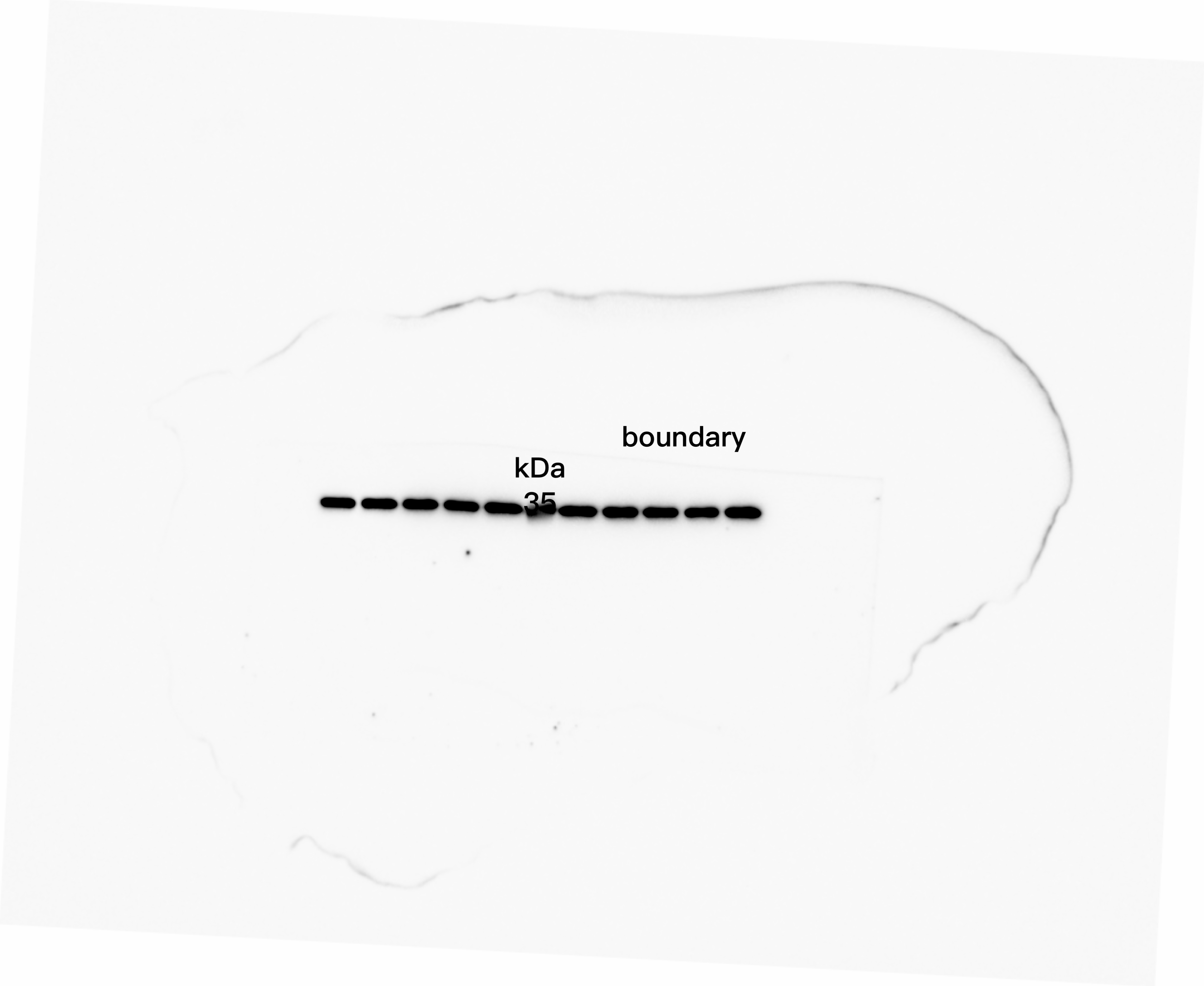

Supplement: Supplementary file 4 — Source data [file 41467_2023_43239_MOESM4_ESM.zip › Source data/Western blot original image /GAPDH (right half).tif]

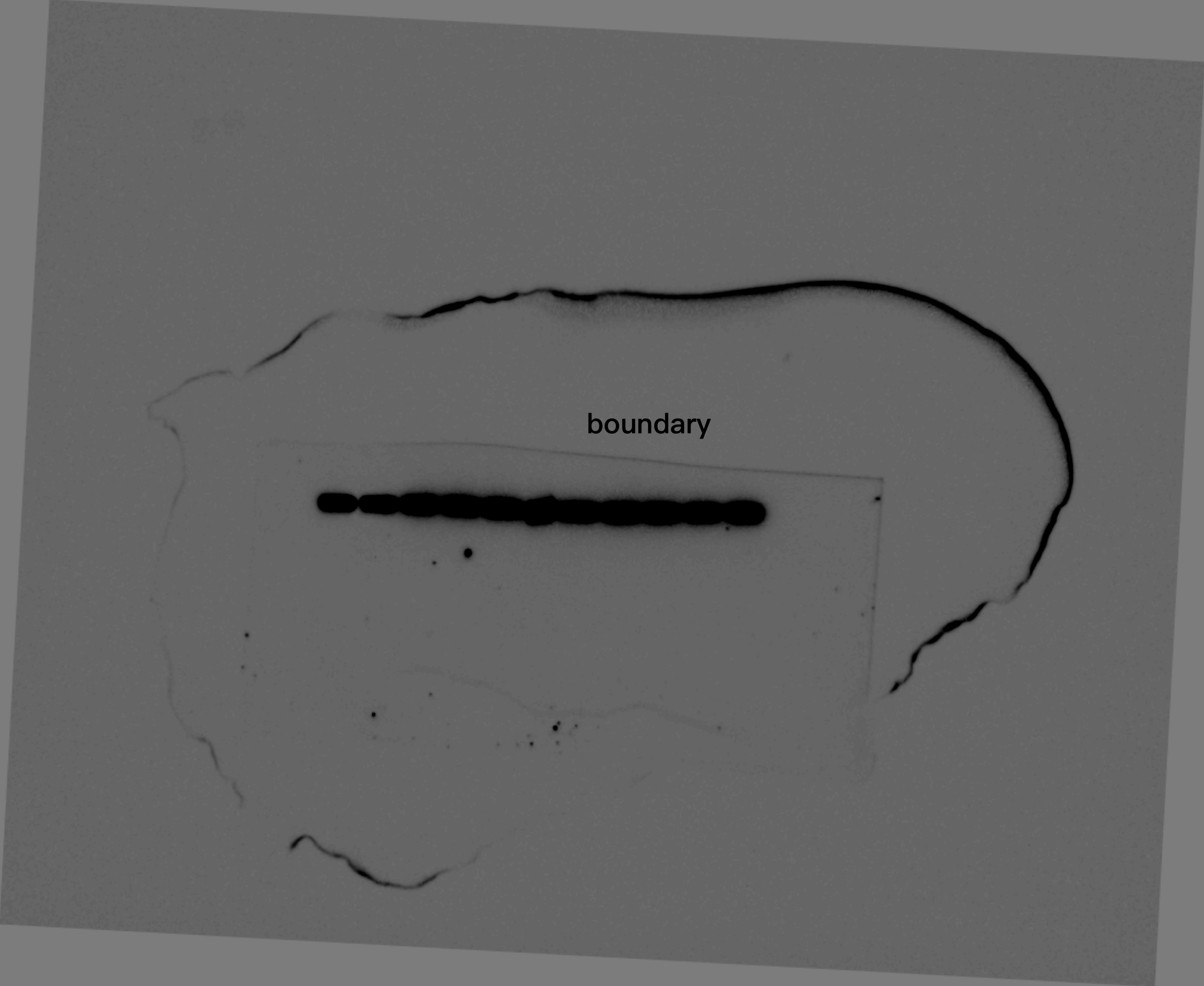

Supplement: Supplementary file 4 — Source data [file 41467_2023_43239_MOESM4_ESM.zip › Source data/Western blot original image /GAPDH (right half)-darkened.tif]

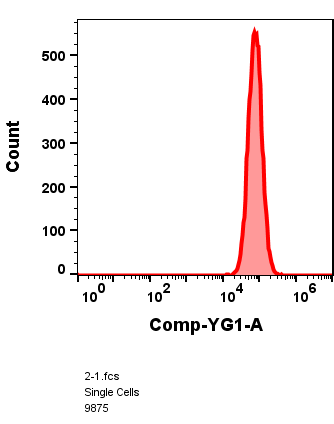

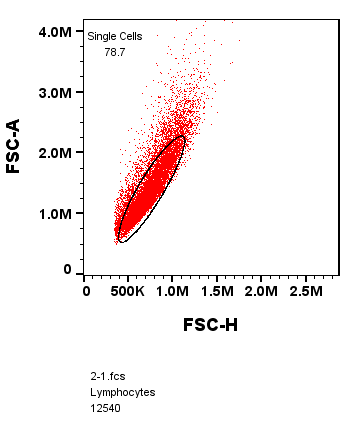

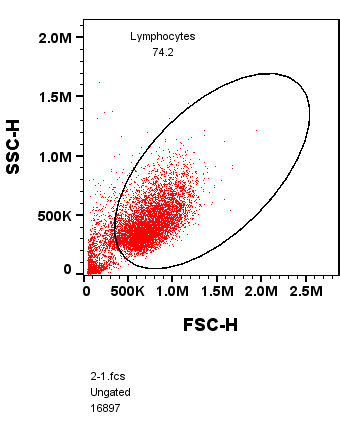

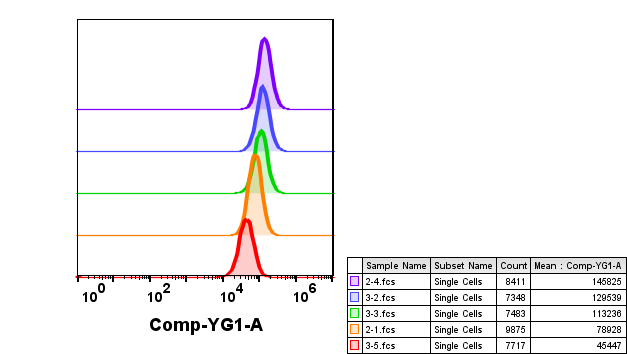

Supplement: Supplementary file 4 — Source data [file 41467_2023_43239_MOESM4_ESM.zip › Source data/Flow cytometry/Flow cytometry in Figure 3.docx]

Figure 4j


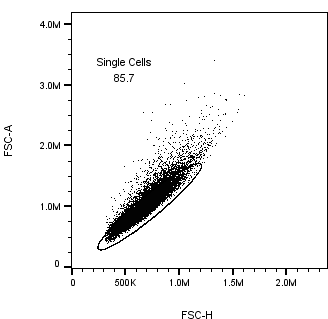

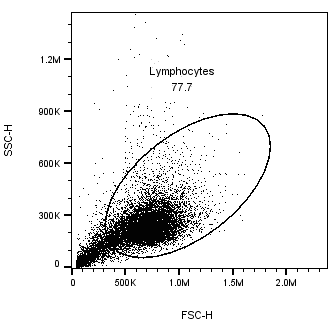

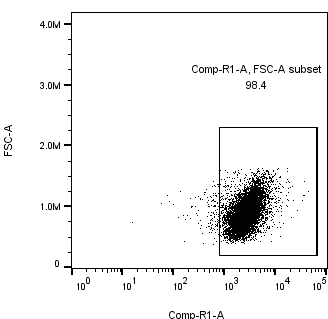

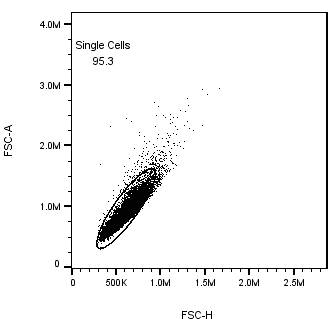

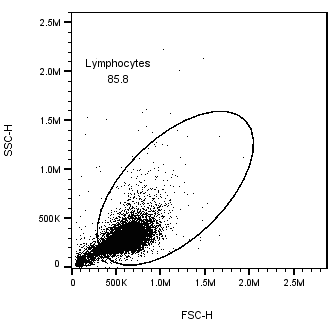

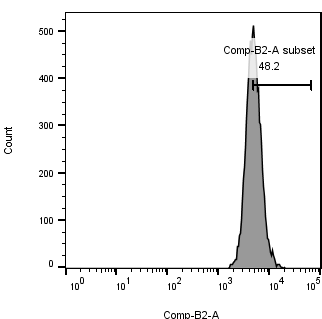


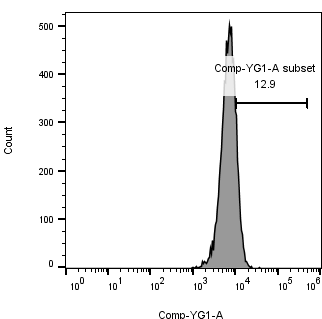

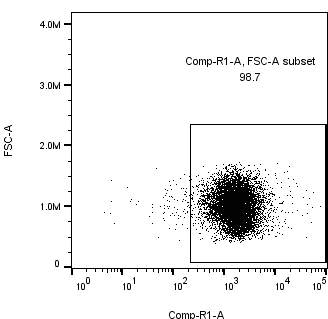


Figure 4k


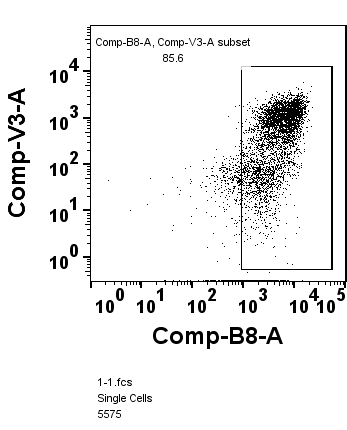

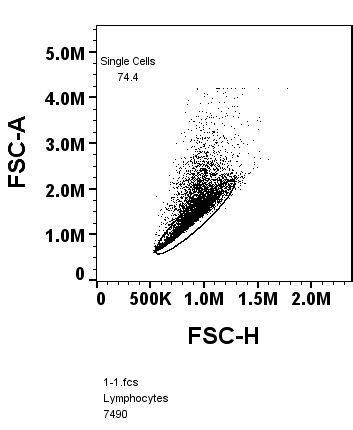

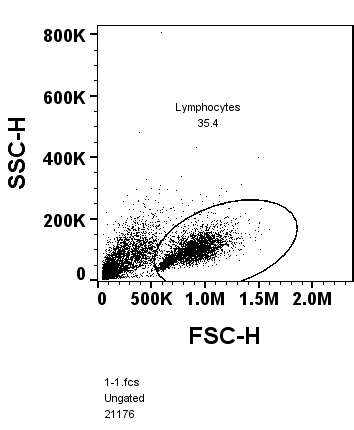


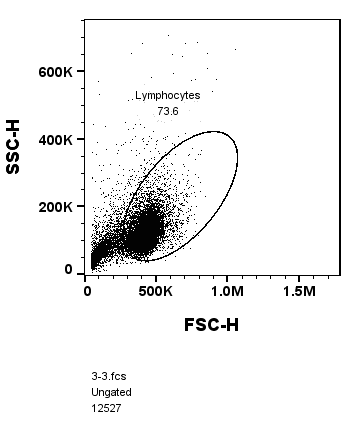

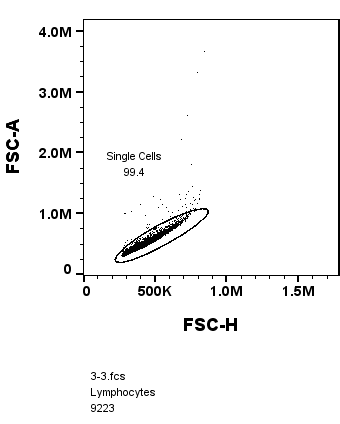

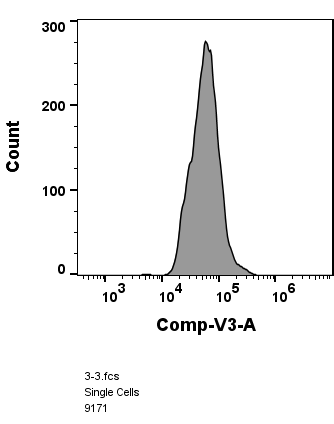

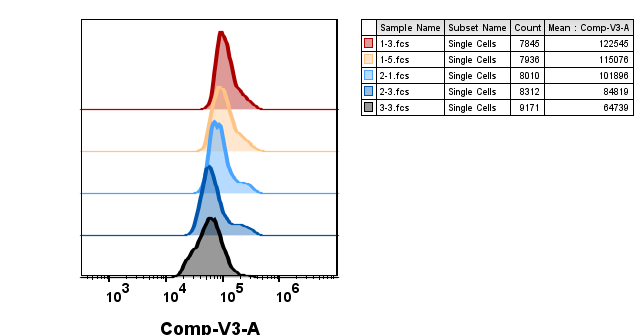
Figure 4i

Supplement: Supplementary file 4 — Source data [file 41467_2023_43239_MOESM4_ESM.zip › Source data/Flow cytometry/Flow cytometry in Figure 4.docx]
